# Spontaneous Escherichia coli Meningitis and Brain Abscess in an Immunocompetent Adult

**DOI:** 10.7759/cureus.28728

**Published:** 2022-09-03

**Authors:** Keesha Jeter, Arun Dang, Aaron Ly, Deepthi Jayasekara

**Affiliations:** 1 Medical School, Western University of Health Sciences, Pomona, USA; 2 Infectious Disease, Emanate Health Queen of the Valley Hospital, West Covina, USA

**Keywords:** e. coli, liver abscess, bilateral limb weakness, ring-enhancing lesions, immunocompetent adult

## Abstract

*Escherichia coli* is widely known to be a common cause of gram-negative bacterial meningitis in neonates and infants but is a rare cause of central nervous system infection in adults. Risk factors for *E. coli* meningitis (e.g., penetrating head trauma or neurosurgery) have been broadly discussed in the literature. Here, we describe a case of spontaneous *E. coli* meningitis with multiple enhancing brain lesions and liver abscess in an immunocompetent adult that presented as generalized weakness.

## Introduction

*Escherichia coli* is a gram-negative rod found in the gastrointestinal tract as part of the normal flora and is typically nonpathogenic [1]. In adults, extraintestinal infections typically occur in the setting of translocation, with the urinary tract being the most common site of infection [1]. *E. coli* is one of the leading causes of neonatal meningitis in the United States, second only to group B *Streptococcus* [2]. However, after the first month of life, *E. coli* meningitis is uncommon, and typically only seen after neurosurgery, trauma, or hepatic cirrhosis [3,4].

Regardless of the pathogen, bacterial meningitis can progress rapidly within a few hours to several days depending on the organism. Although most patients with meningitis present with the classic clinical triad of fever, headache, and nuchal rigidity, others may have signs of altered mental status ranging from lethargy to coma [5,6].

Diagnosis of bacterial meningitis is made via examination of cerebrospinal fluid (CSF). In the case of bacterial meningitis, typical CSF findings include polymorphonuclear leukocytosis, decreased glucose concentration, and increased protein concentration and opening pressure [7]. Bacterial meningitis is a medical emergency, and empiric antibiotics should be initiated within one hour of arrival to the emergency department prior to obtaining the results of the CSF gram stain and culture [6]. Here, we report a case of bacterial meningitis caused by *E. coli* in an adult with multiple brain lesions and liver abscess successfully treated with antibiotics.

## Case presentation

A 37-year-old male with no relevant medical history presented to the emergency department with a one-week history of generalized weakness. He reported a fever and frontal headache partially relieved by acetaminophen. Three days prior to presentation, he had vomiting and three to four episodes of non-sanguineous diarrhea which had resolved. The patient denied any prior history of similar symptoms, weight loss, sore throat, shortness of breath, cough, chest pain, or abdominal pain. He smoked four to five tobacco cigarettes daily and denied alcohol, drug use, or high-risk sexual behavior. He worked as a gardener and reported no recent distant travel.

Upon presentation to the emergency department, he was afebrile. The remainder of his vital signs were as follows: blood pressure of 158/82 mmHg, heart rate of 70 beats/minute, respiratory rate of 17 breaths/minute, and pulse oximetry of 99% on room air. On general examination, he appeared hemodynamically stable and in no acute distress. His heart was at a regular rate and rhythm and his lungs were clear to auscultation. The abdomen was soft, non-tender to palpation, and without masses. Neurological examination revealed the patient was oriented to person, place, and time; his cranial nerves were grossly intact; and there was no nuchal rigidity. Although he appeared weak while ambulating, he had good symmetric strength bilaterally and there were no focal deficits. Over the course of his hospital stay, however, he developed right hemiparesis.

**Figure 1:**
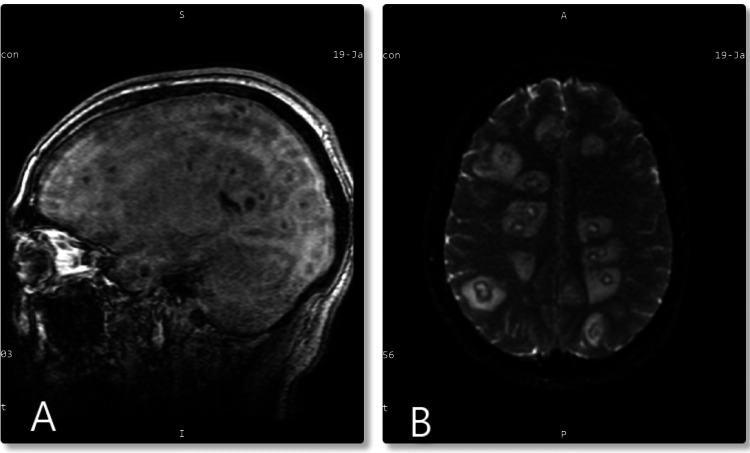
(A) Sagittal view exhibiting cystic brain lesions. (B) Axial diffusion-weighted imaging view showing numerous enhancing cystic lesions throughout the cerebral and cerebellar hemispheres associated with surrounding edema.

Laboratory analysis was performed upon admission (Table 1). The complete blood count showed an elevated white blood cell count of 20,800 k/µL with neutrophilia and platelets of 371 × 10^3^ μ/L. Severe acute respiratory syndrome coronavirus 2, human immunodeficiency virus (HIV), and immunologic studies were negative (Table 2). Chemistry studies showed a sodium of 132 mmol/L. His hepatic function panel demonstrated elevated albumin of 4.9 g/dL, total bilirubin of 1.2 μmol/L, alkaline phosphatase of 174 U/L, and aspartate and alanine transaminases of 46 U/L and 91 U/L, respectively. Due to liver enzyme abnormality, an abdominal ultrasound was ordered. Results showed cholelithiasis without evidence of acute cholecystitis and hepatic steatosis with a complex 6 cm cystic/solid lesion within the right hepatic lobe. A targeted biopsy of the liver lesion showed acute inflammation and abscess tissue with negative acid-fast bacilli (AFB) and periodic acid-Schiff (PAS) stains for microorganisms. Carcinoembryonic antigen (CEA) for liver neoplasm was marginally elevated at 3.5 ng/dL. Chest radiograph showed opacities in the left lung base possibly consistent with developing pneumonia. Computed tomography (CT) of the head without contrast was unremarkable for midline shift, mass lesion, or intracranial hemorrhage. Magnetic resonance imaging (MRI) was recommended after neurosurgical consultation and showed innumerable enhancing cystic lesions throughout the cerebral and cerebellar hemispheres, with the largest measuring up to 13 mm in the right temporal region (Figure 1). Metastatic disease and brain abscesses were considered differential diagnoses. A lumbar puncture was performed with CSF analysis following a negative head CT scan (Table 3). The appearance of the fluid was turbid and bloody with red blood cells of 235,000 cells/µL, white blood cells of 30 cells/µL, polynuclear white blood cells at 74%, increased opening pressure, and total protein of 55 mg/dL. Gram stain showed few white blood cells and no organisms.

**Table 1:** Complete blood count and blood chemistry data.

Laboratory	Patient’s results	Reference range
White blood cells	20.8	4.0–9.6 × 10^3^ μ/L
Hemoglobin	13.0	13.9–16.0 g/dL
Hematocrit	38	40.6–46.4%
Platelet count	371	144–366 × 10^3^ μ/L
Sodium	132	135–146 mmol/L
Potassium	3.9	3.6–5.3 mmol/L
Chloride	101	96–106 mmol/L
Carbon dioxide	24	20–30 mmol/L
Blood urea nitrogen	12	7–22 mg/dL
Creatinine	0.71	0.6–1.30 mg/dL
Glucose	124	65–99 mg/dL
Calcium	9.3	8.6–10.4 mg/dL
Total creatine kinase	32	46–171 U/L
Troponin I	<0.002	0.00–0.045 ng/mL
Total bilirubin	1.2	0.3–1.2 mg/dL
Aspartate transaminase	46	<34 U/L
Alanine transaminase	91	10–49 U/L
Alkaline phosphatase	174	46–116 U/L
Albumin	4.9	3.2–4.8 g/dL

**Table 2:** Immunologic studies.

Laboratory	Patient’s results	Reference range
Immunoglobulin G subclass 4	24.9	4–86 mg/dL
Immunoglobulin E	358.8	<100 IU/mL
% CD4 cells	44	30–61%
Absolute CD4 count	1,096	490–1,740 cells/µL
CD4/CD8 ratio	1.71	0.86–5.00
% CD8 cells	26	12–42%
Absolute CD8 count	641	180–1,170 cells/µL
Carcinoembryonic antigen	3.5	0–2.5 ng/dL

**Table 3:** Cerebrospinal fluid analysis.

Laboratory	Patient’s results	Reference range
Appearance	Turbid	Clear
Color	Bloody	Colorless
Gram stain	2 + (few) white blood cells. No organisms seen. 1 + (rare) red blood cells	
White blood cell count	30	0–5 cells/µL
Red blood cell count	23,560	<0 cells/µL
Mononuclear white blood cells %	26	15–60%
Polynuclear white blood cells %	74	0–6%
Glucose	65	40–70 mg/dL
Total protein	55	15–45 mg/dL

The patient was empirically started on intravenous piperacillin/tazobactam and azithromycin in the emergency department and infectious disease was consulted. Azithromycin was discontinued and intravenous micafungin was started pending the results of the CSF culture. The CSF culture ultimately grew *E. coli*, and he was then transitioned to intravenous ceftriaxone and metronidazole. Broad-spectrum antibiotics were continued due to brain and liver abscesses. Viral and fungal etiologies were considered and serological studies were ordered, both of which were negative (Table 4). In addition, stool testing for ova and parasites was ordered and collected. The patient received a transesophageal echocardiogram to exclude cardiac vegetations and septic emboli, which was negative. By hospital day 15, his symptoms had improved to near baseline and he was discharged on intravenous ceftriaxone and oral metronidazole for an additional two weeks and advised to follow up in the outpatient clinic.

**Table 4:** Microbiology data. Ab: antibody; Ag: antigen; SARS-CoV-2: severe acute respiratory syndrome coronavirus 2; HSV: herpes simplex virus; PCR: polymerase chain reaction; EIA: enzyme-linked immunosorbent assay

Laboratory	Patient’s results	Reference range
Coccidioides Ab (CF)	<1:2	<1:2
SARS-CoV-2 (PCR)	Negative	Negative
Cryptococcus Ag screen	Not detected	Not detected
Cryptococcus Ag titer	Not detected	Not detected
Cysticercosis Ab	<0.90	<0.90
Echinococcus Ab	Negative	Negative
HSV I DNA PCR	Not detected	Not detected
HSV II DNA PCR	Not detected	Not detected
HIV 1 & 2 Ag/Ab, 4th Gen	Non-reactive	Non-reactive
A. Galactomannan Ag EIA	Not detected	Not detected
Aspergillus index value	<0.50	<0.50 not detected
Blood culture	No growth	No growth

## Discussion

Although *E. coli* is one of the most common causative organisms in neonatal meningitis, it is a rare phenomenon in immunocompetent adults. Among other gram-negative bacilli causing adult bacterial meningitis, *Klebsiella pneumoniae*, *Pseudomonas aeruginosa*, and *Acinetobacter *are the most common [3]. Most reported cases of *E. coli* meningitis occur in a nosocomial setting after head trauma or neurosurgical procedures [8]. Spontaneous *E. coli* meningitis occurs even more infrequently, except in patients with comorbidities [9]. Common risk factors include chronic alcoholism, cirrhosis, HIV infection, chronic obstructive pulmonary disease, immunosuppressive drugs, and diabetes mellitus [10]. Our patient did not demonstrate any of the aforementioned risk factors for *E. coli* meningitis.

The most common symptoms of meningitis include fever, headache, neck stiffness, nausea, and vomiting. The clinical triad of fever, altered mental status, and neck stiffness were reported in only 25% of cases of *E. coli* meningitis [9]. However, signs of meningeal inflammation may be absent [11]. Roos et al. found that greater than 75% of patients may present with a decreased level of consciousness [12]. In our case, the diagnosis of *E. coli* meningitis was surprising. He was a healthy immunocompetent adult male who presented with one week of generalized weakness, fever, headache, and only a few days of vomiting and diarrhea that resolved prior to presentation to the emergency department. The lack of the classic triad of meningitis and right hemiparesis prompted further clinical investigation.

MRI of the brain was performed and showed multiple brain abscesses. After neurosurgical consultation, the differential diagnosis suggested either a pyogenic abscess or metastatic disease. On a review of the literature, community-acquired *E. coli* is associated with brain abscesses [13]. Given the patient’s history of gastrointestinal disturbances and eating food from street vendors, a parasitic infection was considered. Cases of *E. coli* sepsis in the setting of nematodal hyperinfection due to *Strongyloides stercoralis* with perforation through the intestinal wall leading to dissemination have been reported, especially in transplant or heavily immunocompromised patients with risk factors [14,15]. Stool analysis for ova and parasites, including serologies for cysticercosis and echinococcus, was ordered and the results were negative.

Along with a head CT scan, evaluation of the CSF is crucial in the diagnosis of meningitis. Typical CSF findings for bacterial meningitis include the following three components: increased protein levels, decreased glucose concentration, and pleocytosis, primarily polymorphic leukocytes [16]. The CSF analysis in our patient showed elevated protein and polynuclear leukocytosis with a normal glucose concentration. Given the patient was from California and presented with a persistent headache along with associated nausea and vomiting, fungal meningitis due to coccidioides, tuberculosis, and histoplasmosis was also considered. However, fungal cultures for CSF and blood were negative. Viral meningitis was also included in our differential.

The abdominal ultrasound showing a cystic-solid hepatic lesion was also an interesting finding in our patient. Although the differential included cystic neoplasm, the biopsy result was consistent with a hepatic abscess. Multiple cases of concomitant liver and ring-enhancing brain abscesses due to *Klebsiella *have been reported, especially in immunocompromised patients or those with risk factors [17,18]. However, there have been few reports of liver and brain abscesses due to *E. coli* described in the literature. While hospitalized, he was started on metronidazole to treat the hepatic abscess which was continued outpatient for four weeks.

The standard empiric treatment of *E. coli* meningitis generally involves a third-generation cephalosporin (e.g., ceftriaxone or cefotaxime), which is efficacious in treating gram-negative pathogens. A three-week course of intravenous antibiotic therapy is recommended for gram-negative bacillary meningitis [6]. Because the CSF culture for our patient showed sensitivity to ceftriaxone, he was continued on a four-week intravenous course. At subsequent outpatient visits, he continued to improve clinically. Two months after the completion of antibiotics, a follow-up MRI of the brain was performed and showed findings consistent with multiple small treated intracranial abscesses without evidence of associated abnormal enhancement or restricted diffusion.

## Conclusions

Although *E. coli* meningitis is most commonly seen in neonates and in post-neurological procedures in nosocomial settings, spontaneous infections are rare in adults. This case report reveals a unique clinical presentation of spontaneous adult *E. coli *meningitis without the classic clinical triad of fever, altered mental status, and nuchal rigidity in an immunocompetent patient with multiple brain and liver abscesses. Clinicians should consider this differential, especially with a non-classical presentation of adult meningitis.

## References

[1] Cornelissen CN, Hobbs MM (2019). Illustrated Reviews: Microbiology. https://meded.lwwhealthlibrary.com/book.aspx?bookid=2605.

[2] Klinger G, Chin CN, Beyene J, Perlman M (2000). Predicting the outcome of neonatal bacterial meningitis. Pediatrics.

[3] Berk SL, McCabe WR (1980). Meningitis caused by gram-negative bacilli. Ann Intern Med.

[4] Pauwels A, Pinès E, Abboura M, Chiche I, Lévy VG (1997). Bacterial meningitis in cirrhosis: review of 16 cases. J Hepatol.

[5] van de Beek D, de Gans J, Spanjaard L, Weisfelt M, Reitsma JB, Vermeulen M (2004). Clinical features and prognostic factors in adults with bacterial meningitis. N Engl J Med.

[6] Jameson JL, Loscalzo J, Kasper DL, Fauci AS, Longo DL, Hauser SL (2018). Harrison's Principles of Internal Medicine. Harrison's Principles of Internal Medicine.

[7] Roos KL, van de Beek D (2010). Bacterial meningitis. Handb Clin Neurol.

[8] van de Beek D, Drake JM, Tunkel AR (2010). Nosocomial bacterial meningitis. N Engl J Med.

[9] Bodilsen J, Brouwer MC, Kjærgaard N, Sirks MJ, van der Ende A, Nielsen H, van de Beek D (2018). Community-acquired meningitis in adults caused by Escherichia coli in Denmark and The Netherlands. J Infect.

[10] Bichon A, Aubry C, Dubourg G, Drouet H, Lagier JC, Raoult D, Parola P (2018). Escherichia coli spontaneous community-acquired meningitis in adults: a case report and literature review. Int J Infect Dis.

[11] Durand ML, Calderwood SB, Weber DJ, Miller SI, Southwick FS, Caviness VS Jr, Swartz MN (1993). Acute bacterial meningitis in adults. A review of 493 episodes. N Engl J Med.

[12] Roos KL, Tyler KL (2018). Acute meningitis. Harrison's Principles of Internal Medicine.

[13] Rau CS, Chang WN, Lin YC (2002). Brain abscess caused by aerobic Gram-negative bacilli: clinical features and therapeutic outcomes. Clin Neurol Neurosurg.

[14] Gomez JB, Maque Y, Moquillaza MA, Anicama WE (2013). E. coli meningitis presenting in a patient with disseminated Strongyloides stercoralis. Case Rep Infect Dis.

[15] Rahim S, Drabu Y, Jarvis K, Melville D (2005). Strongyloidiasis: a mistaken diagnosis and a fatal outcome in a patient with diarrhoea. Trans R Soc Trop Med Hyg.

[16] van de Beek D, Brouwer M, Hasbun R, Koedel U, Whitney CG, Wijdicks E (2016). Community-acquired bacterial meningitis. Nat Rev Dis Primers.

[17] Sun R, Zhang H, Xu Y, Zhu H, Yu X, Xu J (2021). Klebsiella pneumoniae-related invasive liver abscess syndrome complicated by purulent meningitis: a review of the literature and description of three cases. BMC Infect Dis.

[18] Wu C, Han S, Baydur A, Lindgren B (2021). Klebsiella brain abscess in an immunocompetent patient: a case report. J Med Case Rep.

